# Predicting *F*_v_ */F*_m_ and evaluating cotton drought tolerance using hyperspectral and 1D-CNN

**DOI:** 10.3389/fpls.2022.1007150

**Published:** 2022-10-18

**Authors:** Congcong Guo, Liantao Liu, Hongchun Sun, Nan Wang, Ke Zhang, Yongjiang Zhang, Jijie Zhu, Anchang Li, Zhiying Bai, Xiaoqing Liu, Hezhong Dong, Cundong Li

**Affiliations:** ^1^ State Key Laboratory of North China Crop Improvement and Regulation/Key Laboratory of Crop Growth Regulation of Hebei Province/College of Agronomy, Hebei Agricultural University, Baoding, China; ^2^ College of Mechanical and Electrical Engineering, Hebei Agricultural University, Baoding, Hebei, China; ^3^ Institute of Cereal and Oil Crops, Hebei Academy of Agriculture and Forestry Sciences, Shijiazhuang, China; ^4^ Cotton Research Center, Shandong Key Lab for Cotton Culture and Physiology, Shandong Academy of Agricultural Sciences, Jinan, China

**Keywords:** chlorophyll fluorescence parameter *F_v_/F*_m_, high-throughput measurement, cotton, drought tolerance, hyperspectral, one-dimensional convolutional neural network

## Abstract

The chlorophyll fluorescence parameter *F_v_/F_m_* is significant in abiotic plant stress. Current acquisition methods must deal with the dark adaptation of plants, which cannot achieve rapid, real-time, and high-throughput measurements. However, increased inputs on different genotypes based on hyperspectral model recognition verified its capabilities of handling large and variable samples. *F_v_/F_m_* is a drought tolerance index reflecting the best drought tolerant cotton genotype. Therefore, *F_v_/F_m_* hyperspectral prediction of different cotton varieties, and drought tolerance evaluation, are worth exploring. In this study, 80 cotton varieties were studied. The hyperspectral cotton data were obtained during the flowering, boll setting, and boll opening stages under normal and drought stress conditions. Next, One-dimensional convolutional neural networks (1D-CNN), Categorical Boosting (CatBoost), Light Gradient Boosting Machines (LightBGM), eXtreme Gradient Boosting (XGBoost), Decision Trees (DT), Random Forests (RF), Gradient elevation decision trees (GBDT), Adaptive Boosting (AdaBoost), Extra Trees (ET), and K-Nearest Neighbors (KNN) were modeled with *F*_v_ */F*_m_. The Savitzky-Golay + 1D-CNN model had the best robustness and accuracy (RMSE = 0.016, MAE = 0.009, MAPE = 0.011). In addition, the *F*_v_ */F*_m_ prediction drought tolerance coefficient and the manually measured drought tolerance coefficient were similar. Therefore, cotton varieties with different drought tolerance degrees can be monitored using hyperspectral full band technology to establish a 1D-CNN model. This technique is non-destructive, fast and accurate in assessing the drought status of cotton, which promotes smart-scale agriculture.

## Introduction

Cotton (*Gossypium hirsutum* L.) is an important cash crop cultivated globally. Drought is major abiotic stress (Cruz de Carvalho, 2008), whose high frequency reduces the average productivity of major crops by up to 50% globally (Lamaoui et al., 2018). According to the World Food and Agriculture Organization, global food output losses caused by drought during the past decade amount to USD 30 billion (Zhang et al., 2021a). Cultivating drought-resistant varieties is not only important for resistance against frequent droughts but also an important current breeding goal. Drought-resistant varieties strongly tolerate drought, with moderate drought stress stabilizing the yields (Wang et al., 2018).

Breeding and screening drought-resistant varieties are usually complex and time-consuming since it depends solely on breeder expertise. Many relevant reports of the classification methods for different genotypes also exist, which mostly focus on fluorescence scanning, protein electrophoresis, deoxyribonucleic acid (DNA) molecular markers (Zhang et al., 2012), the determination of relative water content, net photosynthesis, stomatal conductance electron transfer rate, photochemical quenching, chlorophyll a/b ratio, plant height, and leaf area (Zou et al., 2020). The high-throughput method has gradually become an important technique for selecting drought-resistant varieties from numerous varieties. Drought significantly decreases the leaf water potential, followed by partial leaf stomatal closure, increased leaf temperature, and reduced photosynthetic efficiency (Najafi et al., 2007; Ahmed et al., 2013). Chlorophyll fluorescence kinetic parameters reflect leaf light energy absorption, transformation, transmission, and distribution characteristics (Hikosaka and Tsujimoto, 2021). The maximum photochemical quantum yield (*F*_v_ */F*_m_) in the chlorophyll fluorescence kinetic parameters represents the maximum light energy conversion efficiency in the photosystem II complex (PSII) reaction center. Thus, drought tolerance indicators have subsequently been developed to evaluate the drought adaptability of different plant genotypes. *F*_v_ */F*_m_ positively correlates with drought degree (Zou et al., 2020). Therefore, *F*_v_ */F*_m_ provides valuable information for evaluating plant physiological changes under drought stress (Lang et al., 2018), hence an efficient drought tolerance index in selecting the best drought tolerant cotton genotype. Measuring the crop drought *F*_v_ */F*_m_ is feasible. However, it requires manual measurements and analysis, a 20–30 min plant adaptation period in the dark, which has low efficiency and requires a heavy workload; hence cannot meet plant phenotype analysis needs, such as high flux, automation, and real-time measurement. Therefore, high-throughput evaluation for screening drought-resistant cotton varieties by *F*_v_ */F*_m_ warrants further studies. Rapid and efficient methods for screening cotton varieties must be developed by combining high-throughput phenotype methods and drought-resistant variety screening (Shakoor et al., 2017; Feng et al., 2019). This study focused on an accurate and robust prediction of drought-resistant varieties among different cotton genotypes, from small to large spatial scales.

Hyperspectral remote sensing performs fast, non-destructive, and economical data collection. Compared to conventional remote sensing, it produces a large amount of spectral information and has a high resolution and strong spectral continuity. It determines the optimal wave width and effective band from large hyperspectral datasets to obtain the best inversion effect (Yao et al., 2013). In addition, it comprehensively and accurately reflects the inherent spectral characteristics and differences between plants. Compared to the traditional identification method, this technology shortens the analysis time and reduces the material crop consumption, such as wheat (Mahesh et al., 2008; Choudhary et al., 2009), rice (Wang et al., 2015), cotton (Carreiro Soares et al., 2016), and grape (Zhao et al., 2018). Using hyperspectral data to monitor plant growth and development is based on plant spectral characteristics. Based on the spectral reflectance in different wavelength ranges, the spectral index provides a high crop parameter inversion accuracy. The vegetation color, cell structure, and water content determine most plant spectral characteristics. Thus, its successful application depends on a full understanding of the interaction between light and plant matter from the cellular to the canopy scale, the interpretation of reflectance data from different sources and related leaf spectral diversity. However, elucidating the interaction between drought and chlorophyll structural characteristics, cell structures, water, visible light, and near-infrared and short-wave infrared regions is a major challenge due to the inability to separate *F*_v_ */F*_m_ from a series of other traits.

Large data volumes and the diversity of analysis methods for hyperspectral data often lead to large data problems (Montesinos-López et al., 2017); hence, advanced algorithms are required for parsing to generate physiological parameters evaluation models. With rapid agricultural artificial intelligence developments (Lu et al., 2017), excellent feature extraction and data inference abilities, and deep learning (DL) algorithms have attracted attention in constructing crop parameter inversion models combined with hyperspectral data (Shah et al., 2019). Machine learning (ML) methods, such as CatBoost, LightGBM, XGBoost, decision trees, Random Forests (RF), Gradient lifting trees (GBDT), adaboost, ExtraTrees, and K-Nearest Neighbor (KNN), are promising for extracting spectral features related to drought resistance by converting original data into new features (Khan et al., 2020). ML usually performs well on a sample-specific basis but loses generalizability when implemented on new data sets with different feature spaces and distributions of different plant species and growth conditions. DL is a new machine learning research field. It was developed to establish and simulate human brain neural networks for analytical learning, and simulates the mechanism of data interpretation in the brain. Thus, it is an unsupervised learning method (Durai and Shamili, 2022; Khan et al., 2022). It derives from artificial neural network research, and its multi-layered perceptron, with multiple hidden layers, which differs from machine learning. Unlike machine learning, DL has the input, hidden, output layers, and an accepting layer. One-dimensional convolutional neural networks (1D-CNN) are one of the most effective and popular deep learning models. It has the advantage of high recognition accuracy (Ghosal et al., 2018) and provides more general and robust leaf biochemical character retrieval. The network framework includes a convolution, pooling, and full connection layer used for feature extraction, compression, and classification, respectively. Convolutional Neural Networks (CNN) are used in many fields, such as weed and pest identification (Ding and Taylor, 2016), plant disease and stress diagnosis (Ghosal et al., 2018), and agricultural image segmentation (Xiong et al., 2017). Therefore, 1D-CNN has a good developmental history and an advantage in physiological parameter evaluation.

Many studies have used hyperspectral models to analyze and screen crop varieties. For example, Miao et al. (2018) introduced the t-SNE model, pretreated by Procrustes analysis (PA), into the field of hyperspectral imaging (HSI) to classify 800 grains of eight waxy maize varieties. Yu et al. (2021) combined DL and neural networks to classify 18 okra varieties. However, in most studies, the prediction results are based on the spectral information of a single growth stage. Combining the data of each growth stage achieves a higher prediction accuracy. As far as we know, research on screening drought resistant cotton varieties based on hyperspectral reflectance and deep learning at various growth stages has not yet been reported. Therefore, a 1D-CNN regression model with reflectance and *F*_v_ */F*_m_ is crucial to screen drought resistant varieties among the different cotton genotypes.

In this study, we aimed to explore the feasibility of *F*_v_ */F*_m_ based on 1D-CNN fitting to evaluate drought resistance among cotton genotypes by screening drought-resistant cotton varieties using hyperspectral and deep learning. The *F*_v_ */F*_m_ and spectral reflectance of 80 cotton genotypes were measured at the flowering, boll setting, and boll opening stages under drought stress. We hypothesized that deep learning with strong interpretation and stability could be used to interpret the specific spectral responses of drought-resistant cotton genotypes, mainly the leaf reflectance in different genotype diversity and environmental change datasets. The specific objectives were: (1) To compare and analyze the full spectral data and the Successive Projections Algorithm (SPA) dimension reduction data; (2) To compare 1D-CNN with Categorical Boosting (CatBoost), Light Gradient Boosting Machine (LightBGM), XGBoost, DT, RF, Gradient elevation decision trees (GBDT), Adaptive Boosting (AdaBoost), Extra Trees (ET), and K-Nearest Neighbors (KNN); (3) To determine whether *F*_v_ */F*_m_ prediction is feasible for screening cotton drought resistant varieties through cluster analysis. Based on Savitzky-Golay (S-G) and 1D-CNN model coupling, an *F*_v_ */F*_m_ evaluation model was created, and a model update strategy was proposed to improve accuracy and robustness.

## Materials and methods

### Plant materials

Eighty cotton cultivars widely cultivated in the Yellow River Basin and the lower reaches of the Yangtze River across different timelines were analyzed in this study, as shown in **Supplementary Table 1**.

### Experimental design and treatments

**Figure 1:**
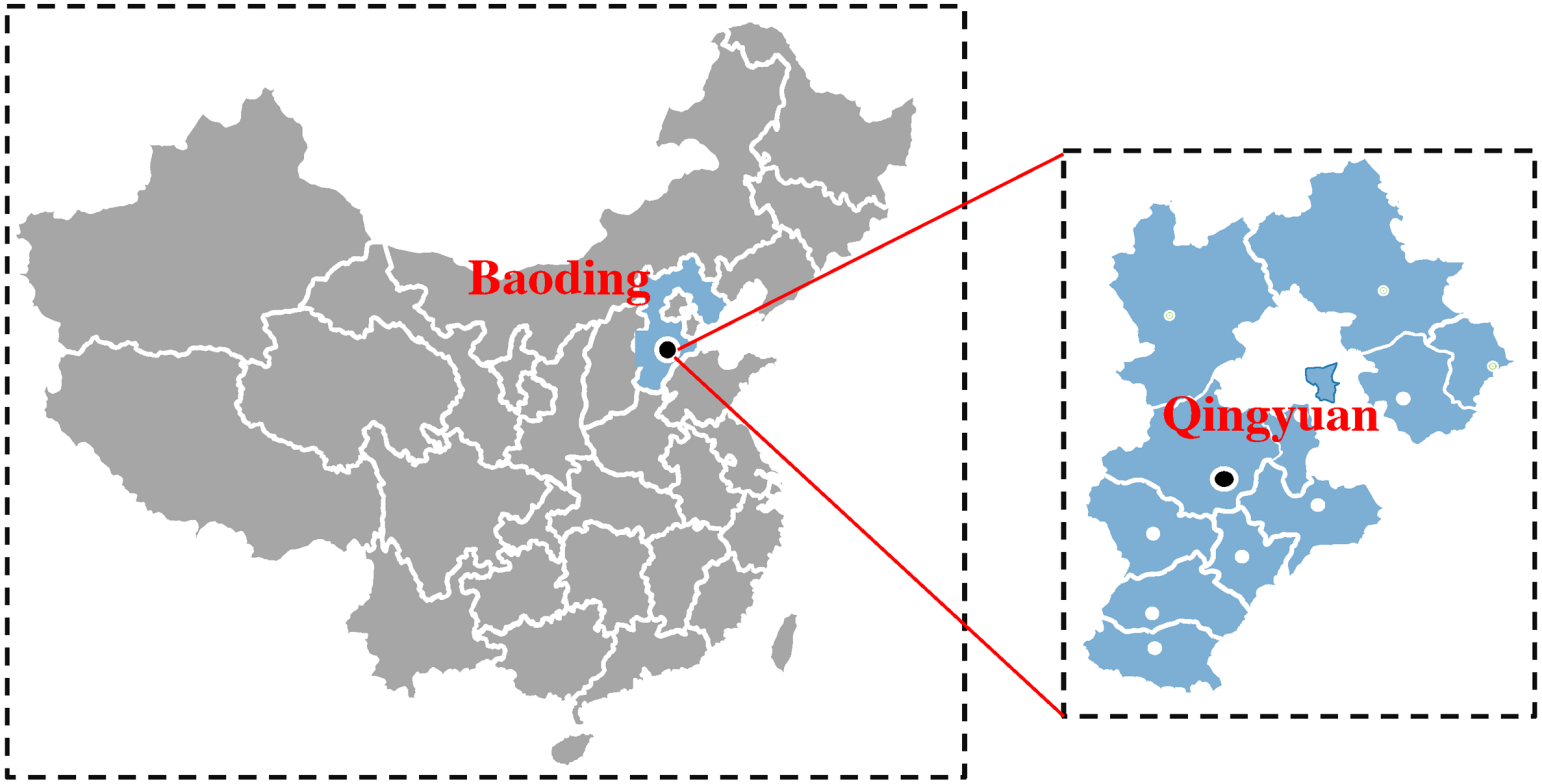
A sampling plot in Qingyuan District, Baoding, Hebei province. The red dot represents the sampling point.

The experiment was conducted in a cotton field at Qingyuan experimental station of Hebei Agricultural University (38.85° N, 115.30° E, Baoding City, Hebei, China) from April to October 2021. The site information (Qingyuan Experiment Station) is presented in **Figure 1**. The study location has a temperate continental monsoon climate, with an average annual average temperature of 13°C and 2700 sunshine hours. The annual average precipitation is 532 mm, with about 60% of the precipitation from July to August. The experiment was laid out in a randomized complete block design (**Supplementary Figure 1**). The experiment had two drought stress levels based on the soil relative water contents (SRWC), including CK (well-watered, 75 ± 5% SRWC serving as the control) and DS (drought stress with 45 ± 5% SRWC) (Gao et al., 2020; Xiao et al., 2020). There were 160 plots per treatment replicated three times totalling 480 plots. The SRWC was monitored by time domain reflectometry (TDR, TRIME TDR series soil moisture meter, IIMKO Company, German) and then watered to maintain the SRWC within the appropriate ranges using micro-sprinkler irrigation.

Selected cotton seeds were sown on 24 April 2021. Four to five seeds were manually sown per hill using the hill-dropping seeding method, with a planting density of 5 plants m^-2^ and a row spacing of 48 cm. Next, mulching was done with a plastic film along the rows. The seedlings were thinned to one vigorous stand per hill upon germination at the two true-leaf stages (Zhang et al., 2021c). Drought stress treatment was induced at the third true-leaf stage. Each plot received 450 kg ha^-1^ of compound fertilizer containing 15% N, 15% P_2_O_5_ and 15% K_2_O as base fertilizer, and 150 kg ha^-1^ urea (46% N) was top-dressed at flowering. In addition, pest control, weed control, chemical control, and plant pruning were performed according to local agronomic practices. The soil texture based on the USDA soil classification standards of the tested soil at different soil layers in the cotton field is shown in **Supplementary Table 2**.

An electrically powered rain-out shelter was used to protect the plants against receiving precipitation. A rain sensor automatically controlled the rain-out shelter switch. The shelter closed automatically in the event of rain and opened as soon as the rain stopped. Thus, as described previously, any possible interference of natural precipitation with the waterlogging experiment was avoided.

### Determination of indices and methods

Leaf hyperspectral, *F*_v_ */F*_m_, RWC and LWC were measured on 6 July 2021 (flowering stage), 14 August 2021 (boll setting stage), and 17 September 2021 (boll opening stage). Three representative plants were randomly selected from each plot. The specific determination of indices and methods was as follows:

### Hyperspectral data collection

Based on the HR-1024i spectrometer (SVC, USA), the instrument blade clamp light source was used to measure the leaf surface reflection spectrum. The spectrometer had a measurement range of 350–2500 nm and a total of 1024 channels. The spectral resolution was 3 nm, and the sampling interval was 0.6 nm. To ensure a full spectrometer probe view field on leaf samples under the sun, the spectrometer sensor probe was vertically oriented downward, about 0.7 m from the cotton canopy top, and the field angle was set at 25 degrees. White board correction was carried out before each measurement to reduce error. The measurements were carried out in sunny, cloudless, windless, or low wind speed weather, between 10:00 am and 2:00 pm. Three representative, uniform, and pest-free plants were selected from each test plot to measure the reflection spectrum of the top four and fully developed leaves after topping. Before each measurement, the dust on top of the cotton leaves was wiped off to ensure the leaf surfaces were kept clean. Four sample points per leaf were selected, and their average was used as the leaf reflection spectrum. Measurements were taken once per month for three consecutive months. After field spectrum measurements, the top leaf for each plant was marked on its underside and labelled with a serial number for subsequent *F*_v_ */F*_m_ measurements to ensure consistency. The detailed determination method and leaf selection are presented in **Supplementary Figure 2**.

### Chlorophyll fluorescence content

A portable FMS-2 fluorometer (Hansatech, King’s Lynn, UK) was used to measure the chlorophyll fluorescence characteristic parameter *F*_v_ */F*_m_ for newly developed, inverted leaves. Leaf initial (*F_o_*_)_ and maximum fluorescence (*F*_M_) were measured from 0:00 am to 2:00 am. The maximum photochemical quantum yield was then calculated as *F*_v_ */F*_m_ = (*F*_m_-*F*_o_)/*F*_m_ (Bilger and Björkman, 1990).

### Root water contents and leaf water contents

Three plants were selected and uprooted from each plot. Next, their roots and shoots were separated, and the fresh weights were determined. The roots and shoots were then dried 80°C to a constant weight to determine the dry weights. Finally, the water content was calculated as follows:


(1) $$Water\;content(\%)=\frac{Fresh\;weight-Dry\;weight}{Fresh\;weight}\times 100$$

### Calculation of drought resistance coefficient

The average *F*_v_ */F*_m_ was measured to calculate the drought tolerance coefficient as described by Mwadzingeni et al., 2016.


(2) $$Drought\;tolerance\;coefficient\;of\;F_{v}/F_{m}(\%)=\frac{Average\;value\;of\;F_{v}/F_{m}\;DS}{Average\;value\;of\;F_{v}/F_{m}\;CK}$$

*F*_v_ */F*_m_ is the maximum photochemical quantum yield, *CK* is the normal conditions, *DS* indicates drought stress.

### Spectral pretreatment and characteristic wavelength screening

#### Extraction, reflectance, and spectral pretreatment

The first step was to superimpose and match all spectral curves. In the second step, S-G first-order smoothing was used to eliminate spectral noise and reduce the influence of environmental background interference due to the spectral mutation of the instrument (del Amor et al., 2020). The third step was to remove the file header from the processed data, generate raw data, and save it as a TXT text file. The fourth step was calculating the averages of spectral data and generating spectral data for each ground object type. The fifth step was to interpolate the obtained data because the whiteboard reflectance band did not match the spectral band of each ground object type. The final step was to select the fourth data column (percentage) in the file and multiply the whiteboard reflectance according to the reflectance formula described by Zhao et al., 2022. This test adopted the vertical measurement method using the following formula:


(3) $$Rt=\frac{L}{Lr}Rr$$

*Rt* is the reflectivity of the measured object, *Rr* is the reflectivity of the standard version, *L is* the measured value of the measuring object, *Lr* is the standard value of the instrument.

#### SPA filter characteristic wavelength

A total of 1440 data groups were recorded. The SVC HR (overlay) software was used to extract the wavelength and reflectivity of each sample, and MATLAB was used to perform SPA on all spectra data to extract the characteristic wavelengths. Relevant source code can be found online https://blog.csdn.net/weixin_43637490/article/details/118468559.

### Model development

#### One dimensional convolutional neural network (1D-CNN)

**Figure 2:**
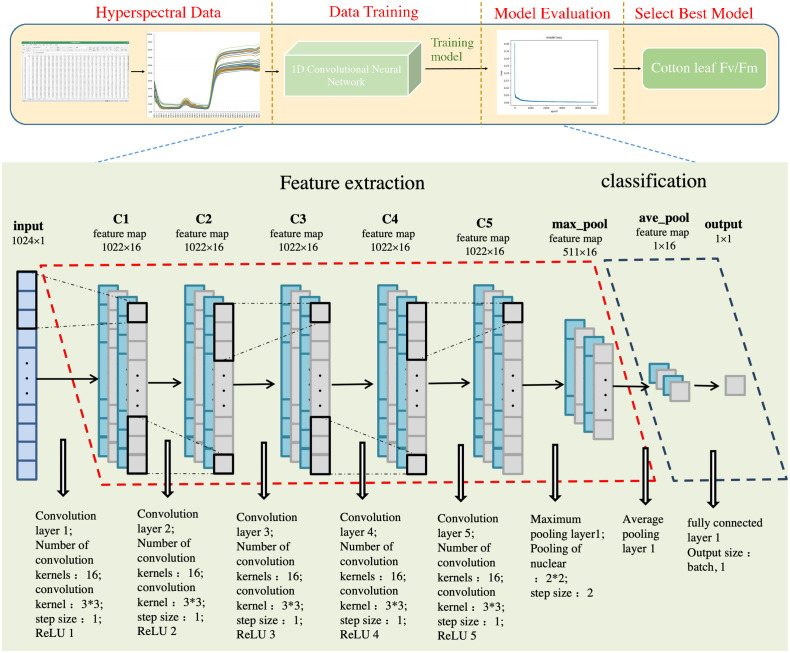
Flow chart illustrating 1D-CNN data processing. The training set accounts for 75% and the test set 25%. A matrix was created with 72 rows, 1024 columns, and a dimension added to the training set channel. Another matrix was created with nine rows, 1024 columns, one channel number, zero elements, and a dimension was added to the test set channel. Finally, an empty deep learning model named “model” was defined. Next, five one-dimensional convolution layers were added (corresponding to five ReLU activation functions), including 16 convolution cores measuring 3 x 3 and a step length of one. The first maximum pool layer was added, and the pool core size was 2 x 2, and the step distance was two. The first global average pooling layer was then added, yielding the first full connection layer; the output size was (batch, 1). The RMSProp optimizer was then defined with a learning rate of 0.001, the gradient decay rate of 0.9, the fuzzy factor was zero, and the learning rate decay rate was zero. The MAE loss function and RMSProp optimizer were then used. The framework of the in-depth learning model was integrated, and data was transferred to the defined model by training 5000 epochs; the amount of data in each batch was 5. Finally, the table was given a title, and the image displayed. The test code is the same as the above training code.

1D-CNN modeling was used to screen the spectral information of cotton drought-resistant genotypes. The main reasons were as follows: (1) the CNN network analyzed one-dimensional data (leaf spectral information) well. (2) It was able to advance the nonlinear mode from the data. (3) It allowed hierarchical spectral data processing to support feature abstraction and extraction. CNN is one of the best algorithms in deep learning, which can be divided into one-, two-, and three-dimensional. 1D-CNN is a classical deep neural network with high robustness, similar to 2D, with a local connection and weight-sharing characteristics. 1D-CNN was selected to adapt to the nature of spectral data (that is, the spectral reflectance had a one-dimensional data structure) to allow the convolution operation to extract the learning features of patterns. A convolutional neural network is usually used for image recognition, target detection, and classification (Fukushima, 1980). 1D-CNN also performs well in time series prediction and data fitting. In contrast, 2D-CNN is mainly used for image and text recognition, and 3D-CNN is for video recognition and medical applications. Due to its unique structure, CNN processes network structure data characteristics well, effectively solving the data processing difficulties caused by other factors (Liang et al., 2020). The hierarchy proposed in this study includes an input layer, multiple hidden layers (convolution, activation, and pooling layer), and the composition of a full connection (dense) and output layer (**Figure 2**) (Zhang et al., 2021b):

The convolution layer functions to extract input data features. Different convolution kernels are equivalent to different feature extractors. The main feature is the use of weight sharing and local connections. The operation of one-dimensional convolution is shown in formula (3):


(4) $$y^{i}=f(\Sigma_{i}k^{ij}*x^{i}+b^{j})$$

where * represents convolution operation, *y^i^* is the i^th^ output characteristic diagram, *x^i^* is the i^th^ input characteristic diagram, *k^ij^* is the convolution kernel used in the layer convolution calculation, and *b^j^* is the offset of the j^th^ characteristic diagram.

For the nonlinear transformation of features extracted from CNN and dense layers, the output of these layers and extracted features were activated using the corrected linear unit (ReLU) function (Cui and Fearn, 2018) (formula (4). The nonlinear activation function ReLU has a low computational cost and fast convergence speed. Its formula is:


(5) $$R(x)=max(0,x)if\{\begin{array}{c} x<0;R(x)=0\\ x\geq 0;R(x)=x \end{array}$$

where *x* is the feature of CNN or dense layer calculation.

The pooling layer was abstracted as statistical information extraction to reduce dimensionality and minimize array dimension based on maintaining the original characteristics. The convolution layer significantly reduces the number of network connections. Adding a pooling layer after a convolution layer avoids overfitting to a certain extent. The pooling layer effectively reduces the number of neurons, making the network invariant to small local morphological changes, which creates a larger receptive field. Two types of common pooling functions are recognized: maximum pooling (taking the maximum value of all neurons in a region) and average pooling (taking the average value of all neurons in a region), expressed as in formula (5) and (6), respectively:


(6) $$p^{l}=max[a^{l}]$$

(7) $$p^{l}=\frac{1}{k}\sum a^{l}$$

where *p* is the characteristic matrix obtained by pooling, *l* is the characteristic graph width, and *a* is the characteristic matrix after convolution layer activation. The maximum and average pooling values calculate the maximum and average values in the adjacent rectangular area, respectively, and location-independent information can be obtained through the maximum pooling value.

The full connection layer is similar to the relationship between one layer and the next layer in the feed-forward network, in which each node of the upper layer and the nodes of the next layer have a weight connection. It is mainly used to complete the final prediction. Each output neuron of the full connection layer is connected to the neuron in the upper layer, and the input characteristics are combined after the activation function is used to output the prediction results. For the prediction problem, the output layer gives the probability value of the prediction category. Its output is given by formula (7):


(8) $$\delta_{i}=f(w_{i}p_{i}+b_{i})$$

where *i* = 1, 2, and k; δ is the *i* ^th^ output, with a total of K outputs; *w*_i_ and *b*_i_ are the weights and thresholds of the *i* ^th^ neuron, respectively; and *f* (*x*) is the activation function.

In this study, a vector that extracted 1024 spectral features was constructed as the input layer, the *F*_v_ */F*_m_ prediction value was used as the output layer (usually, the input vector length is larger than the convolution kernel length), and the hidden layer included 1D-CNN with five convolution layers and two pooling layers (**Figure 2**). The spectral data was convoluted, and the convolution filter (also known as the kernel) was used to extract the feature map. The scaler variable was used to accept the entire data normalization process for the following anti-normalization. Data were subsequently normalized in Excel by subtracting the mean and dividing by the variance.

The number of hidden layers, the number of feature maps in each layer, the CNN kernel size, the pool and step size, and the regularization parameters are all adjustable and were optimized by experience to obtain the best value. The optimized architecture specification is presented in **Figure 2**. Additionally, the proposed architecture was developed as a common architecture for multiple scenarios and case studies (multiple independent data sets), while the existing architecture was evaluated separately on a single data set. The training data set in the CNN model developmental stage was randomly divided into two sub-datasets, calibrated and validated. During feed-forward and backpropagation, these batches were sequentially fed into the network. Once all batches were entered into the model (training era), the validation data set was used to evaluate model efficiency and accuracy on unknown samples. The model was trained on 6 July 2021 (flowering stage), 14 August 2021 (boll setting stage), and 17 September 2021 (boll opening stage) to ensure sample calibration and verification convergence.

### Machine learning models

For a more comprehensive model performance and accuracy comparison, nine machine learning algorithms, including CatBoost, LightBGM, XGBoost, DT, RF, GBDT, AdaBoost, ET and KNN, were used for modeling and comparative analysis using 1D-CNN.

CatBoost is a decision tree-based model consisting of an open source software library developed by Hancock (2020) with categorical features in a special way. LightGBM is a distributed gradient boosting framework based on a decision tree algorithm, which supports single-machine multi-threading and multi-machine parallel computing, to quickly process massive data (Meng et al., 2016). XGBoost is an additive model that optimizes only the sub-model in the current step in each iteration (Chen and Guestrin, 2016). DT is a non-parametric supervised learning tool with a tree structure composed of four elements: decision nodes, program branches, state nodes, and probability branches (Sarker et al., 2020). RF is a typical bagging algorithm in ensemble learning (Breiman, 2001), that randomizes the use of variables (columns) and data (rows) to generate many classification trees and then summarizes the results of the classification trees. GBDT was developed by Friedman (2001), and builds on each tree, learning the residual (negative gradient) of the sum of all previous tree conclusions (Kriegler and Berk, 2010). AdaBoost is an algorithm for constructing strong classifiers as a linear combination of simple weak classifiers (Freund and Schapire, 1997; Wang et al., 2022). ET is directly divided using random features and random thresholds on random features (Geurts et al., 2006; Ahmad et al., 2018). KNN was proposed by Cover and Hart (1967) and is not limited to a fixed number of parameters (Guo et al., 2003).

### Model evaluation

To evaluate model performance, leaf samples from each data set were sorted, and 75% of the samples were used as the training data set and the remaining 25% as the test data set. In deep learning, the loss function is used to find errors or deviations in the learning process. However, the loss function uses the same metrics as the training process, which differs in value, to evaluate the performance of the generated model to ensure species fairness in the training and testing data sets (Burnett et al., 2021). Therefore optimization is a key step in comparing prediction and loss functions to optimize input weights. During model training, full-spectrum data is used as input, and model accuracy and loss are recorded simultaneously. The network parameters are fine-tuned based on the results. Therefore, the determination coefficient (R^2^), Root Mean Square Error (RMSE), Mean Absolute Percentage Error (MAPE) and Mean Absolute Error (MAE) are selected to accurately evaluate test results (Ibrahim et al., 2021).

Set the predicted value to: $\hat{y}=\{\hat{y_{1}},\hat{y_{2}},\cdot \cdot \cdot \hat{y_{n}}\}$ And the true value to *y* = {*y*_1_, *y*_2_,· ··, *y_n_*}.R^2^ is the determination coefficient. The higher the model R^2^, the higher the accuracy, and the better the fitting effect. The formula is as follows:


(9) $$R^{2}=1-\frac{\sum_{i=1}^{n}{\left(y_{i}-{\hat{y}}_{i}\right)}^{2}}{\sum_{i=1}^{n}{\left(y_{i}-\bar{y}\right)}^{2}},\in [1,0]$$

RMSE is the root mean square error, the difference between the predicted and actual values. The smaller the model RMSE value, the better the model prediction. The calculation formula is as follows:


(10) $$RMSE=\sqrt{\frac{1}{n}\sum_{i=1}^{n}{\left(y_{i}-{\hat{y}}_{i}\right)}^{2},\in [0,+\infty )}$$

MAPE is the mean absolute percentage error; a statistical index used to measure prediction model accuracy. The smaller the model MAPE value, the higher the prediction model accuracy. The calculation formula is as follows:


(11) $$MAPE=\frac{100\%}{n}\sum_{i=1}^{n}\left|\frac{\hat{y}i-yi}{yi}\right|$$

MAE is the mean absolute error, which is the average of the absolute error between the real and predicted values. It accurately reflects the predicted error value. The larger the model MAE value, the greater the error, indicating a lower prediction model accuracy. The calculation formula is as follows:


(12) $$MAE=\frac{1}{n}\sum_{i=1}^{n}|yi-\hat{y}i|$$

where *n* is the number of samples, *yi* is the true values of cotton PH or AGB, $\hat{y}i$ is the predicted values of cotton PH or AGB, and $\bar{y}i$ is the average of the PH or AGB true values.

## Results

### Effects of drought stress on *F*_v_ */F*_m_ in cotton leaves

**Figure 3:**
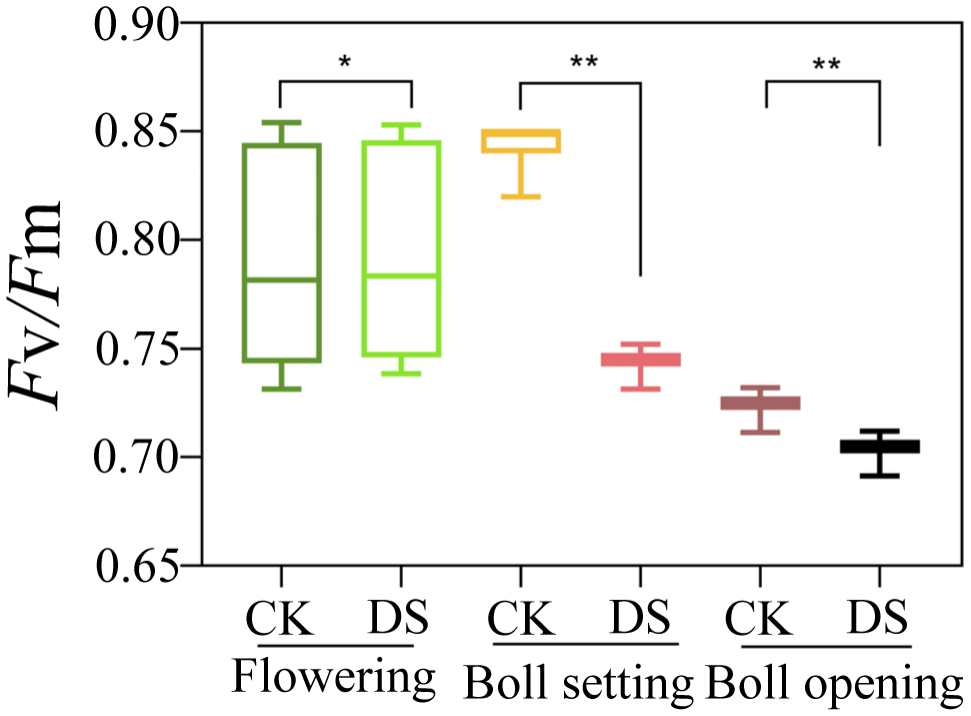
Effects of drought stress on *F*_v_ */F*_m_ at the flowering, boll setting, and boll opening stages. CK, normal conditions; DS, drought stress. * and ** indicate significance at the 0.05 and 0.01 probability levels, respectively.

Generally, when comparing drought stress effects on *F*_v_ */F*_m_ (**Figure 3**), statistical differences were observed among the flowering, boll setting, and boll opening stages (*p* ≤ 0.05). The DS and CK were initially increased and then decreased in the three cotton growth stages. DS treatment significantly reduced *F*_v_ */F*_m_ (*P*< 0.05), by 2%, 12%, and 3% across the three stages, respectively.

### Correlation between *F*_v_ */F*_m_ and RWC, and LWC

**Figure 4:**
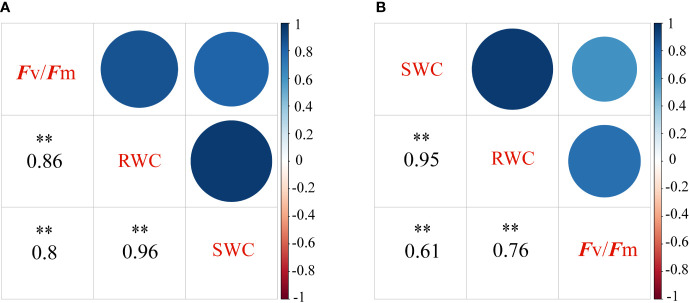
Spearman correlation coefficients matrix and the corresponding 95% confidential levels between *F*_v_ */F*_m_ and the water contents in the roots and leaves. Under normal conditions **(A)** and under drought stress **(B)**, respectively. The significance level of correlations is indicated as follows: **P< 0.01. *F*_v_ */F*_m_, the maximum photochemical quantum yield; RWC, Root water contents; LWC, leaf water contents.

Correlation analysis between *F*_v_ */F*_m_ and RWC, and LWC is illustrated in **Figure 4**. The results revealed a significant positive correlation between *F*_v_ */F*_m_ and RWC, and LWC under DS (**Figure 4B**) and CK (**Figure 4A**). Thus, *F*_v_ */F*_m_ significantly positively correlated with drought resistance in cotton. *F*_v_ */F*_m_ was further used as the input in the model to evaluate the drought resistance of cotton.

### Preprocessing of hyperspectral data

**Figure 5:**
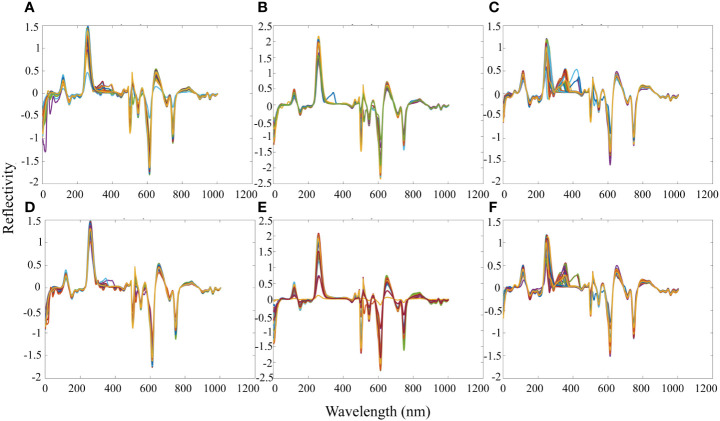
Image analysis of spectral data, preprocessed by Savitzky-Golay, under different drought conditions and cotton growth stages. **(A)** The flowering stage of cotton under normal conditions; **(B)** The boll setting stage of cotton under normal conditions; **(C)** Boll opening stage of cotton under normal conditions; **(D)** The Flowering stage of cotton under drought stress; **(E)** The boll setting stage of cotton under drought stress; **(F)** Boll opening stage of cotton under drought stress.

The spectrum was pretreated to reduce the influence of the external environment, the dark current of the spectrometer and to eliminate baseline drift, light scattering, and spectrum noise. The Savitzky-Golay technology was applied to preprocess the hyperspectral data, eliminating spectral differences (filtering noise and smoothing waveforms) caused by different scattering levels and enhancing spectral and data correlation. The spectral band peaks and valleys were obvious, overlapping peak interference was avoided, and spectral resolution and sensitivity were improved through Savitzky-Golay pretreatment (**Figure 5**).

### Changes in the cotton canopy reflectance spectrum under different conditions

**Figure 6:**
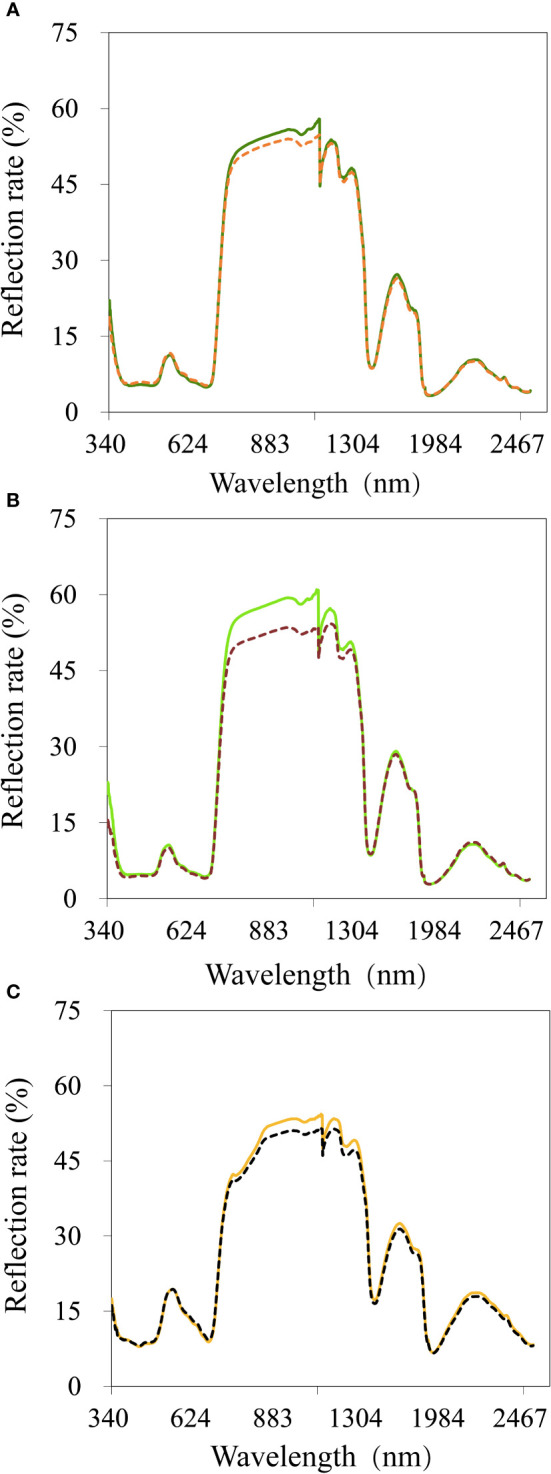
Average reflectance spectra of cotton leaves under different drought conditions and growth stages. **(A)** The flowering stage of cotton under normal conditions and drought stress; **(B)** The boll setting stage of cotton under normal conditions and drought stress; **(C)** The boll opening stage of cotton under normal conditions and drought stress; Each line represents the average value of 240 reflectance spectra of 80 different cotton varieties. Solid lines represent normal conditions; dashed lines represent drought stress.

The cotton canopy spectral reflectance was measured at the flowering (**Figures 6A**), boll setting (**Figures 6B**), and boll opening stages (**Figures 6C**), respectively. The trends for the different varieties at different growth stages were similar, and the differences were obvious under different soil water conditions (**Figure 6**). In the visible light region (350–750 nm), there were two absorption valleys (370–510 and 600–710 nm) and reflection peaks (520–580 nm). The canopy spectral reflectance increased with drought stress, especially at the “green peak”. The higher the soil water content, the better the plant growth, the larger the leaf area index, the higher the chlorophyll content, the stronger the absorption of blue and red light, and the deeper the red valley, leading to an obvious green peak. The opposite scenario leads to a shallower red valley and, thus, a gentler and less obvious curve at the green peak. However, a reflection platform (760–1250 nm) occurred in the near-infrared region (750–1350 nm), where 1000 nm dropped abruptly. Spectral reflectance decreased with drought stress due to cotton cell structural changes, especially in the “near-infrared platform”, where the difference was significant. A lower spectral reflectance occurs under heavy drought stress. The spectral canopy curves under different drought conditions showed similar trends in the other growth stages. The reflectivity showed a downward trend in the short infrared band (1350–2500 nm), and two water absorption bands occurred at the 1450 and 1950 nm bands.

### Full band modeling and analysis and comparative analysis of various modeling methods

To determine the best model algorithm for predicting cotton leaf *F*_v_ */F*_m_, we used the full band and the characteristic wavelengths screened by the SPA algorithm to compare and analyze 1D-CNN, CatBoost, LightBGM, XGBoost, DT, RF, GBDT, AdaBoost, ET, and KNN, respectively. The characteristic wavelengths screened by SPA were inadequate (**Supplementary Table 3**). Thus, only full band modeling results are shown here (specifically, training and test sets; **Table 1**). 1D-CNN, CatBoost, LightBGM, XGBoost, DT, RF, GBDT, AdaBoost, ET, and KNN had a relatively stable model accuracy under the different drought conditions during the flowering stage, but nine of the machine learning algorithms (CatBoost, LightBGM, XGBoost, DT, RF, GBDT, AdaBoost, ET, and KNN) were relatively unstable in estimating *F*_v_ */F*_m_ during the boll setting stage under drought stress. 1D-CNN was also relatively unstable in estimating *F*_v_ */F*_m_ during the boll setting stage under drought stress. However, the 1D-CNN model had the highest accuracy and the best effect in the comprehensive evaluation of cotton drought stress. The flowering stage had the highest accuracy when comparing the predictions and analyses of the various stages. The model was more stable under normal conditions.

**Table 1:** Modeling of drought tolerance at different cotton growth stages with different prediction models.

Prediction model	Conditions	Flowering stage			Boll setting stage			Boll opening stage		
		RMSE	MAE	MAPE	RMSE	MAE	MAPE	RMSE	MAE	MAPE
1D-CNN	CK	0.016	0.009	0.011	0.003	0.005	0.003	0.002	0.005	0.001
	DS	0.010	0.005	0.006	0.006	0.005	0.007	0.002	0.005	0.002
CatBoost	CK	0.021	0.018	2.299	0.001	0.001	0.115	0.003	0.002	0.283
	DS	0.017	0.015	1.849	0.003	0.002	0.315	0.004	0.003	0.454
LightGBM	CK	0.010	0.005	0.629	0.002	0.001	0.167	0.002	0.001	0.162
	DS	0.005	0.003	0.369	0.004	0.003	0.425	0.003	0.002	0.310
XGBoost	CK	0.010	0.003	0.445	0.001	0.001	0.143	0.002	0.001	0.183
	DS	0.007	0.003	0.334	0.005	0.003	0.449	0.003	0.002	0.311
DT	CK	0.010	0.003	0.424	0.002	0.001	0.133	0.002	0.001	0.180
	DS	0.009	0.005	0.636	0.005	0.003	0.405	0.003	0.002	0.275
RF	CK	0.010	0.004	0.528	0.001	0.001	0.089	0.002	0.001	0.177
	DS	0.007	0.004	0.556	0.004	0.003	0.345	0.003	0.002	0.282
GBDT	CK	0.010	0.003	0.390	0.001	0.001	0.086	0.002	0.001	0.174
	DS	0.007	0.004	0.508	0.004	0.003	0.362	0.003	0.002	0.237
AdaBoost	CK	0.010	0.003	0.431	0.001	0.001	0.087	0.002	0.001	0.176
	DS	0.003	0.001	0.171	0.004	0.003	0.343	0.003	0.002	0.313
ET	CK	0.010	0.005	0.608	0.001	0.001	0.086	0.002	0.001	0.157
	DS	0.005	0.003	0.428	0.004	0.002	0.311	0.003	0.002	0.279
KNN	CK	0.038	0.022	2.765	0.001	0.001	0.123	0.003	0.002	0.250
	DS	0.044	0.032	4.011	0.004	0.003	0.424	0.004	0.003	0.365

1D-CNN, One-dimensional convolutional neural network; CatBoost, Categorical Boosting; LightBGM, Light Gradient Boosting Machine; XGBoost, eXtreme Gradient Boosting; DT, Decision Tree; RF, Random Forest; GBDT, Gradient elevation decision tree; AdaBoost, Adaptive Boosting; ET, Extra Trees; KNN, K-Nearest Neighbors; RMSE, Root mean square error; MAPE Mean absolute percentage error; MAE, Mean absolute error.

**Figure 7:**
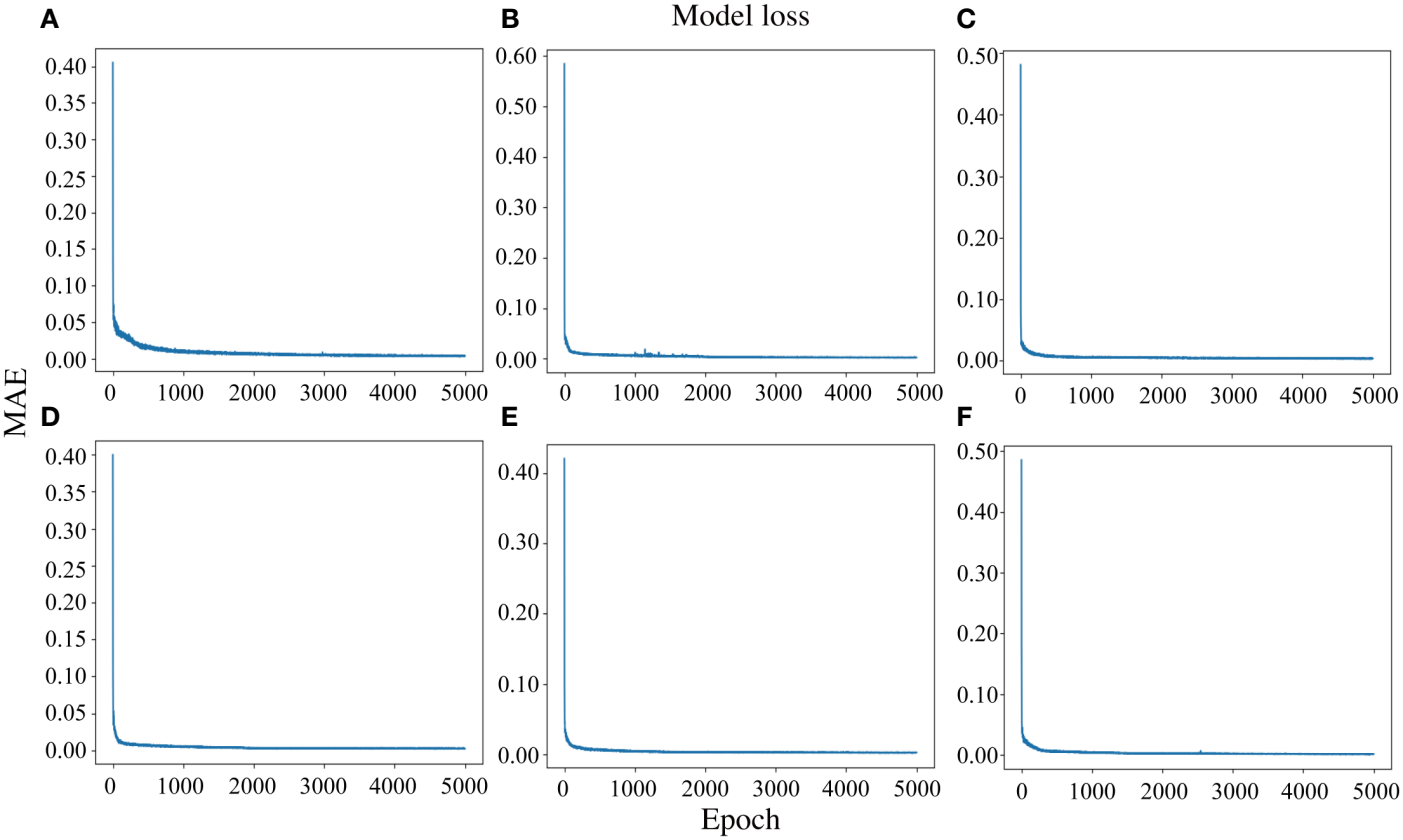
The loss rate curve of the 1D-CNN model with different drought conditions and cotton growth stages. **(A–C)**, Flowering stage, boll setting stage, and boll opening stage under normal conditions, respectively; **(D–F)**, Flowering stage, boll setting stage, and boll opening stage under drought stress, respectively.

Furthermore, the loss function of 1D-CNN was observed to decrease rapidly, and the loss rate was low, which improved the accuracy and reduced diagnosis time, leading to a better diagnosis performance (**Figure 7**).

### *F*_v_ */F*_m_ as predicted from canopy characteristics

**Figure 8:**
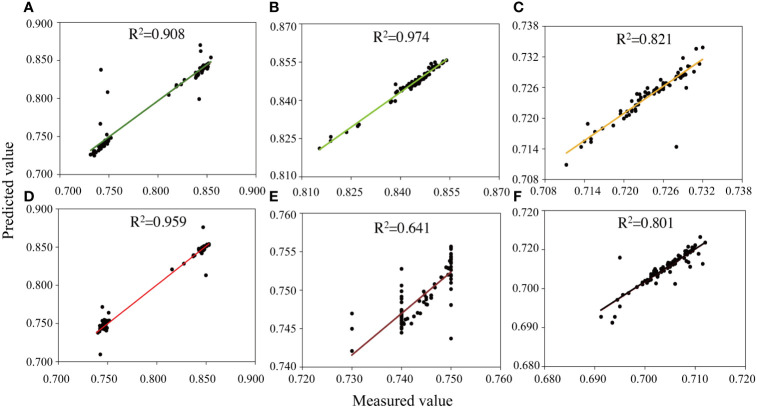
*F*_v_ */F*_m_ predicted and measured values from the test data set under **(A–C)** sufficient water conditions and **(D–F)** water stress conditions. The canopy characteristics input by each model is from **(A, D)** 6 July 2021 (flowering stage), **(B, E)** 14 August 2021 (boll setting stage), and **(C, F)** 17 September 2021 (boll opening stage).

To evaluate the cotton drought tolerance using the spectral features extracted by 1D-CNN, the predicted *F*_v_ */F*_m_ value was determined by 1D-CNN and correlated with the actual value (**Figure 8** **)**. Generally, under sufficient water conditions and drought stress, the correlation between the predicted and measured values was high (R^2^ ≥ 0.641). However, the correlation coefficient was the highest under sufficient water conditions (R^2^ of flowering, boll setting, and boll opening stages were 0.908, 0.974, and 0.821, respectively; Predicted and measured of flowering, boll setting, and boll opening stages were 0.7894 and 0.7923, 0.8467 and 0.8439, 0.7246 and 0.7241, respectively; **Figures 8A-C**). In addition, the correlation coefficient at the flowering stage was the highest among the treatments (R^2^ of CK and DS were 0.908 and 0.959, respectively; Predicted and measured of CK and DS were 0.7894 and 0.7923, 0.7959 and 0.7955; respectively; **Figure 8A, D**).

### Cotton drought tolerance evaluation based on the *F*_v_ */F*_m_ drought tolerance coefficient and cluster analysis

**Figure 9:**
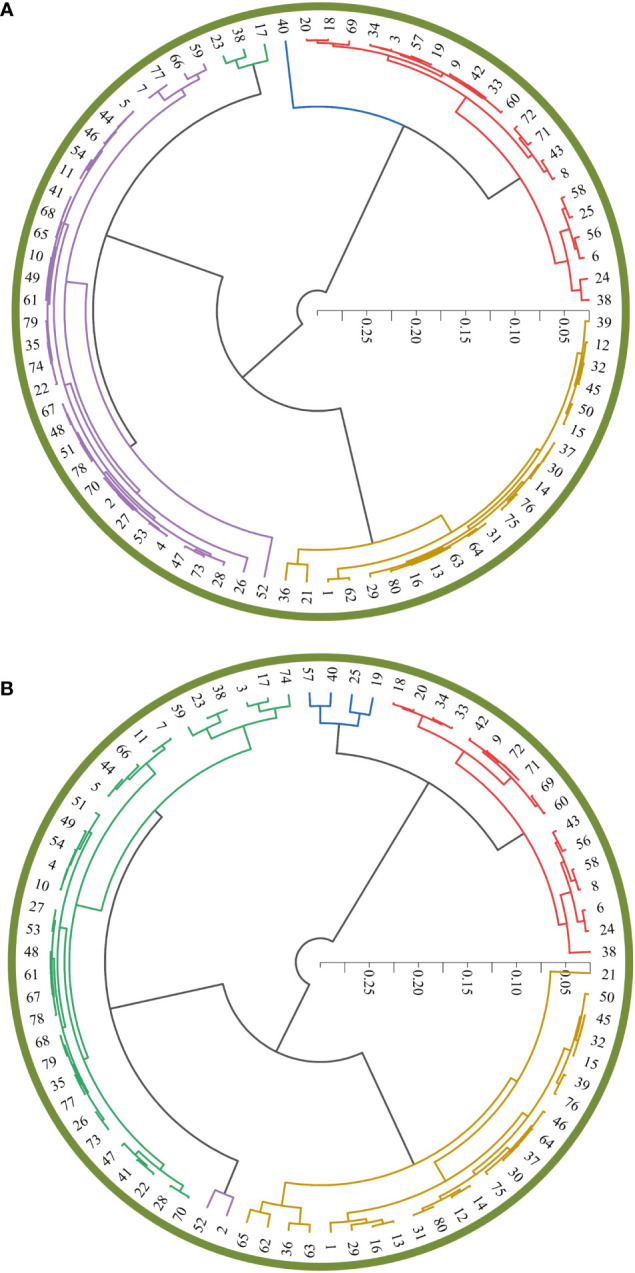
Cluster analysis of the drought tolerance coefficients of the *F*_v_ */F*_m_ measured values **(A)** and predicted values **(B)** for the 80 cotton varieties. 1, Jifeng 554; 2, Jifeng 103; 3, Jifeng 522; 4, Jifeng 908; 5, Jifeng 914; 6, Jifeng 1982; 7, Jifeng 4; 8, 7886; 9, Cangmian 268; 10, Jimian 315; 11, Han 218; 12, Hannong 12; 13, Han 8266; 14, Han 258; 15, Han 686; 16, YM111; 17, Nongda KZ05; 18, Nongdamian 10; 19, Nongdamian 12; 20, Lumianyan 28; 21, Xuzhou 1818; 22, Zhongmiansuo 41; 23, Shandongxiamian11-42; 24, Zhongmiansuo 12; 25, Yumian 19; 26, Ejing 1; 27, Zhongmiansuo 35; 28, Zhongmiansuo 60; 29, Xinshi 71143; 30, Xinza 15; 31, Xinshi 17; 32, GK39; 33, 0 shi; 34, Zhongmiansuo 94A915; 35, Lumianyan 36; 36, DP33B; 37, Guoxinmian01; 38, Guoxinmian02; 39, Guoxinmian03; 40, Guoxinmian05; 41, Hanwu 216; 42, Zhongmian 100; 43, Zhongmiansuo 79; 44, Cangmian 666; 45, Han 6203; 46, Shikang 126; 47, Cang 198; 48, Ji 228; 49, Guoxinmian 9; 50, K836; 51, Lumian 522; 52, Lumian 5172; 53, K638; 54, Guoxin 4; 55, Jifeng1187; 56, Jifeng 1458; 57, Jifeng 103; 58, Jifeng 914; 59, Jifeng 965; 60, MH335223; 61, Guoxinmian 11; 62, Zhongmiansuo 17; 63, Chunbeibao; 64, Zhongmiansuo 60;65, CG3020-3; 66, Jimian 2016; 67, Ji 1518; 68, Jihang 8; 69, Jimian 262; 70, Ji 178; 71, Ji 172; 72, Yuzaomian 9110; 73, Dexiamian 1; 74, Jicai 6913; 75, Zhongmiansuo 23; 76, Zhongmiansuo 50; 77, Ji668; 78, Zhibao 86-1; 79, Jimian 958; 80, Jifeng 1271.

Since the above fitting effect was the highest at the flowering stage, the drought tolerance coefficient was used to evaluate cotton drought tolerance. We clustered the *F*_v_ */F*_m_ and predicted value drought tolerance coefficients through cluster analysis, thereby highlighting the varieties with strong drought tolerance (**Figure 9**). We assumed that the higher drought tolerance coefficients for predicted or measured *F*_v_ */F*_m_ values indicated enhanced drought resistance. The predicted *F*_v_ */F*_m_ classification was similar to the manual measurement classification (**Figures 9A, B** **)**. The top ten drought tolerant varieties obtained through cluster analysis and evaluation of the measured drought tolerance coefficients were: 38, 24, 6, 56, 25, 58, 8, 43, 71, and 72 (**Figure 9A**). The top ten drought tolerant varieties predicted were 38, 24, 6, 56, 25, 58, 8, 43 71, and 72 (**Figure 9B**). The *F*_v_ */F*_m_, drought tolerance coefficient, can be more reliably evaluated from remote sensing data.

## Discussion

This study revealed a high correlation between *F*_v_ */F*_m_, RWC and LWC; thus, *F*_v_ */F*_m_ can be used as a direct indicator for evaluating the drought resistance of cotton. In addition, *F*_v_ */F*_m_ and 1D-CNN models are good at predicting the inversion process of physiological and biochemical cotton indicators and hyperspectral data. The models also achieved the expected effects, and this method can quickly and nondestructively evaluate cotton drought tolerance.

### Relationship between measurement parameters under drought stress

Cotton flowering and boll setting stages are extremely sensitive to soil water content and are important for adequate yield, which significantly declines under stress (Bange et al., 2004; Pettigrew, 2004). Therefore, this study evaluated the drought resistance of cotton varieties by investigating the effects of drought stress on cotton plants at the flowering, boll setting, and boll opening stages in the field. Leaf photosynthetic structure is an important index to evaluate plant stress resistance and plays a key role in plant growth and metabolism, especially for photosystem PSII (El-Hendawy et al., 2019a). PSII maximum photochemical efficiency (*F*_v_ */F*_m_) has widely been used as an indicator for the early detection of different abiotic stresses (Naumann et al., 2008), which directly reflect crop damage under adverse environments. Under normal environmental conditions, *F*_v_ */F*_m_ is relatively stable, but under adverse environmental conditions, photosynthetic efficiency is limited, and chloroplasts are protected from light damage, thereby significantly reducing *F*_v_ */F*_m_ (Castañeda-Murillo et al., 2022). The findings in this study are consistent with those by Fracheboud (2002), where under drought stress, the *F*_v_ */F*_m_ values of cotton varieties decreased during the growth period. Therefore, *F*_v_ */F*_m_ values have gained interest as a screening tool to study preliminary and indicative responses to the rapid changes in plant photosynthetic status, and to evaluate the irreversible physiological damage caused by drought tolerance.

### Optimizing input variables for the 1D-CNN model is important for hyperspectral inversion of cotton *F*_v_ */F*_m_ prediction and drought tolerance evaluation

Numerous studies have mostly used vegetation index as an input to evaluate the degree of stress (Li et al., 2022). However, current vegetation index information is still limited, and the lack of a stable vegetation index closely related to drought stress may eventually reduce model generalizability. However, several specific spectral indices exist that have considerable potential in accurately estimating relevant parameters. SPA is a forward variable selection algorithm that minimizes vector space collinearity (Araújo et al., 2001). Its advantage lies in its extraction of several characteristic wavelengths from the whole band, which eliminates redundant information in the original spectral matrix when screening characteristic spectral wavelengths (Zhang et al., 2019). It is mainly divided into the following steps: firstly, data is imported under different processes; secondly, the Kennard stone algorithm is used to select samples; finally, SPA is used to select variables for multivariable calibration (Zhao et al., 2022). In this study, we used the MATLAB 2019b software to screen the characteristic spectral reflectance wavelengths of each process by coding a continuous projection algorithm, and only 1–2 sensitive wavelengths were screened under normal conditions at the flowering, boll setting, and boll opening stages. This challenged the establishment of a unified spectral index to estimate potential complex factors. Therefore, to improve relevant parameter prediction accuracy, some studies used the full spectrum wavelength (350–2500 nm) (Hansen et al., 2002; El-Hendawy et al., 2019b).

Interestingly, our study revealed that compared to the screening characteristic wavelengths using the continuous projection algorithm, the *F*_v_ */F*_m_ predictions in the calibration and validation data sets had additional improvements based on the full band 1D-CNN model analysis. The maximum coefficient of determination values (R^2^) and minimum root mean square error values (RMSE) further revealed that the 1D-CNN model, based on data fusion in all conditions, was the most accurate in predicting *F*_v_ */F*_m_. Rasooli Sharabian et al., (2014) reported similar results. Elsayed et al. (2020) also revealed that compared to a single spectral index, a PLSR model based on spectral index data fusion and canopy temperature improved the GY prediction accuracy of barley and wheat under water stress. This study also revealed that the fusion of full band spectral data further improves the *F*_v_ */F*_m_ prediction accuracy of cotton drought tolerance under different conditions. This is because this method can measure potential confounding factors related to environmental conditions. Therefore, it covers all the major physiological plant changes induced by drought stress.

This study supports machine learning and deep learning methods instead of the traditional cotton growth parameter estimation methods. Compared to CatBoost, LightBGM, XGBoost, DT, RF, GBDT, AdaBoost, ET, and KNN, the Fv/Fm remote sensing prediction accuracy inversion model constructed by 1D-CNN was higher and had strong stability. This shows that predicting physiological and biochemical indices and evaluating cotton drought tolerance using hyperspectral technology is feasible. In the field, different varieties have different leaf optical characteristics and canopy structures; thus, spectral interpretation is very complex. Despite these complexities, 1D-CNN achieved high accuracy in independent verification. 1D-CNN has previously been used for image segmentation, weed detection and prediction of other crops (such as rice and soybeans). However, the use of 1D-CNN for many cotton varieties is rarely reported. Based on our experimental process, the 1D-CNN estimation method used many characteristics and can use cotton spectral values directly as input, automatically learning and selecting features from the training data. Compared to traditional machine learning, 1D-CNN local connection, weight sharing, and hierarchical expression ensure that the network model effectively learns corresponding data features from many samples, avoids the complex feature extraction process and does not require manual feature extraction. Therefore, 1D-CNN improves prediction accuracy and reduces workload.

### Possible problems with hyperspectral and 1D-CNN models

Unfortunately, 1D-CNN also has challenges, such as a high square error and high deviation (underfitting), which are mainly caused by inadequate sample number, inconsistent distribution of the training and verification sets, complex network structure (such as 1D-CNN), excessive sample noise interference, poor data quality, and overtraining. From the perspective of variance and deviation, underfitting equates to high training set variance and deviation, which performs well in the training set. Still, it performs poorly in the test and new data sets. Generally, the main methods required to effectively solve overfitting are to increase the data set size, simplify and regularize the model, increase the drop layer, perform feature selection and sparse learning, delete abnormal noise points, use integrated learning methods, and re-clean the data.

From this study, spectral reflectance alone may not be sufficient to identify the most drought-tolerant cotton lines during screening. Therefore more phenotypic information sources are needed to fully clarify the complexity of drought tolerant genotype responses in cotton.

### Influence of time scale differences on model performance

Different time scales and their effects on plant growth must be adopted in agricultural development as a management strategy. The reasons for spectral differences between different time scales are plant growth, phenological development, and environmental changes (Fava et al., 2009; Meerdink et al., 2016; Yang et al., 2016). These differences may be inverted in the relationship between spectra and traits, which is what we detected in the performance of each independent model for the flowering, boll setting, and boll opening stages. Li et al. (2022) constructed six sorghum genotype models of dry and fresh weight using support vector machines on two separate dates and found that the combined model accuracy was higher than each independent model. Compared to the boll setting and opening stages, the flowering stage model was more robust and accurate (RMSE = 0.016, MAE = 0.009, MAPE = 0.011).

We observed that specific time scales affected accuracy. This study showed that the flowering stage accuracy was higher than the other stages. This may be due to vigorous growth of cotton crops during the early stage, rapid leaf area increases, large pigment accumulation in vegetation tissue, metabolic increase, high photosynthetic activity, strong *F*_v_ */F*_m_ absorption, and a gradually enhanced regression equation fitting effect. With the postponement of the cotton growth period and the stress and aging of cotton plants in the later stages, leaves started losing their green coloration, turned yellow, and gradually withered. The *F*_v_ */F*_m_ content subsequently decreased significantly until the leaves withered and died, unable to absorb light energy, and dry matter accumulation stopped (Silva Benavides et al., 2013), thus, leading to fitting effect deterioration. This is, therefore, the best period to estimate *F*_v_ */F*_m_.

## Conclusion

**Figure 10:**
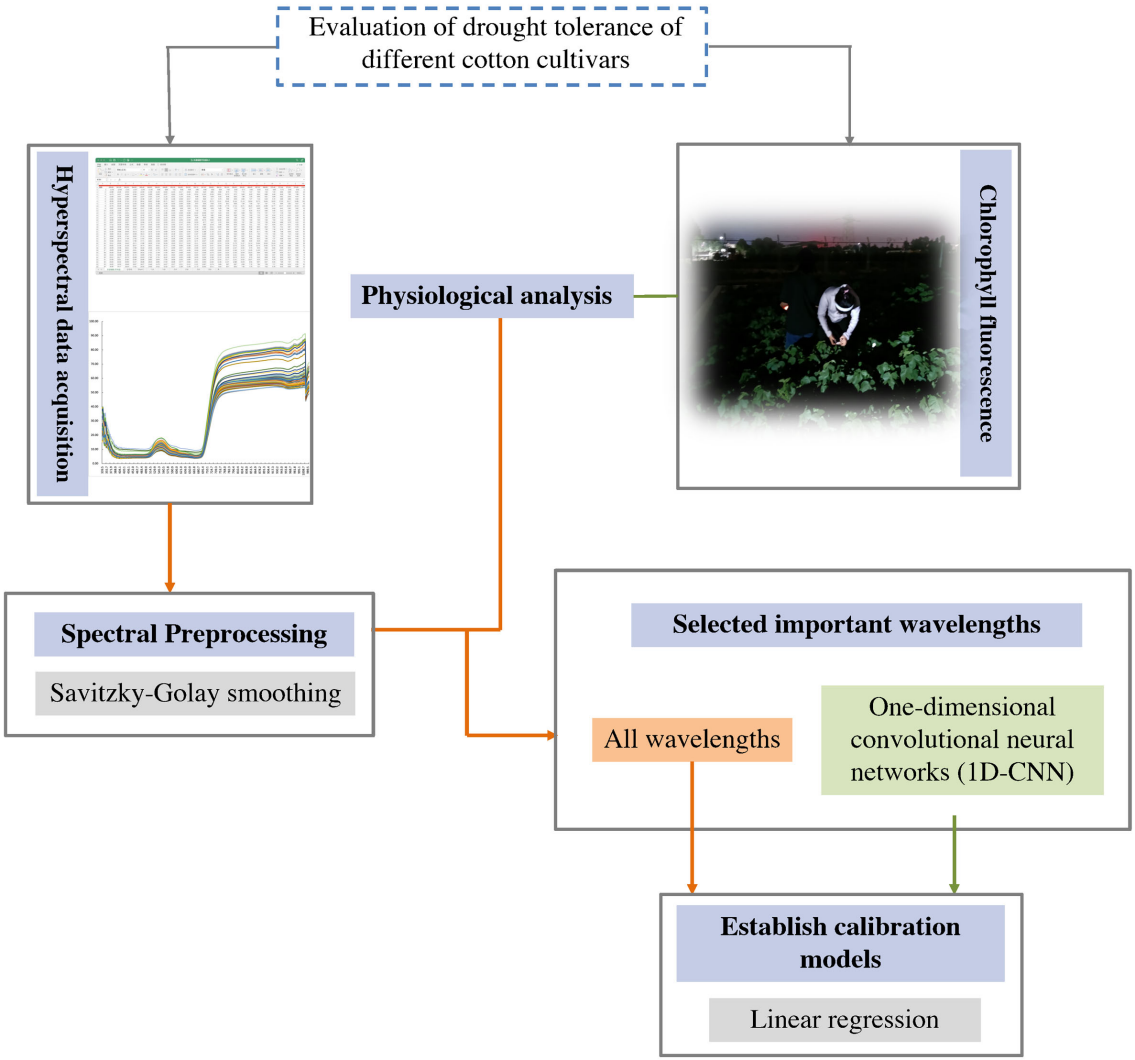
Chlorophyll fluorescence and hyperspectral reflectance approach for detecting drought tolerance in cotton.

Full band spectral data was studied here to predict *F*_v_ */F*_m_ values and to evaluate cotton drought tolerance, (**Figure 10**) showed the workflow. The spectral distribution of the 80 cotton varieties at different growth stages and under different water stress conditions had similar trends. However, their near-infrared band reflectance decreased with drought stress and increased then decreased with growth. Compared to CatBoost, LightBGM, XGBoost, DT, RF, GBDT, AdaBoost, ET, and KNN, 1D-CNN models predicted cotton *F*_v_ */F*_m_ during the three growth stages, implying that 1D-CNN models have higher accuracy and stability in the large-scale data processing. In evaluating cotton drought tolerance, the predicted *F*_v_ */F*_m_ clustering results were similar to manually measured clustering results. Generally, the combined technology of S-G+1D-CNN has been successfully applied to predict cotton variety Fv/Fm values and evaluate drought tolerance. The full spectrum might therefore become an important tool for drought tolerance screening. In this study, it was not necessary to destructively sample all test field indicators, thus greatly reducing cost and time. This accelerated the related processing of phenotypic information for the different varieties and helped to develop a detection system for the high-throughput phenotypic identification algorithm. Therefore, more consideration should be given to spectral data and the computational power of deep learning models to reveal deeper phenotypic information. These models can be used to evaluate and screen out drought-resistant cotton varieties.

## Data availability statement

The raw data supporting the conclusions of this article will be made available by the authors, without undue reservation.

## Author contributions

LL, HD, and CL initiated and designed the research. CG, HS, and XL performed the experiments and collected the data. CG, NW, and AL wrote the code and tested the methods. CG, HS, KZ, YZ, JZ, and ZB analyzed the data and wrote the manuscript. All authors revised and approved the submitted version of the manuscript.

## Funding

This study was supported by grants from the National Natural Science Foundation of China (No. 31871569 and 32172120), Natural Science Foundation of Hebei Province (No. C2020204066), Graduate Innovation Funding Project of Hebei Province (CXZZBS2020089), and the Modern System of Agricultural Technology in Hebei Province (No. HBCT2018040201).

## Acknowledgments

We would like to thank the National Natural Science Foundation of China (No. 31871569 and No.32172120), the Natural Science Foundation of Hebei Province(C2020204066), and the Modern System of Agricultural Technology in Hebei Province (No. HBCT2018040201) for financially supporting our research. Many thanks to MogoEdit (https://www.mogoedit.com) for its English editing during the preparation of this manuscript.

## Conflict of interest

The authors declare that the research was conducted in the absence of any commercial or financial relationships that could be construed as a potential conflict of interest.

## Publisher’s note

All claims expressed in this article are solely those of the authors and do not necessarily represent those of their affiliated organizations, or those of the publisher, the editors and the reviewers. Any product that may be evaluated in this article, or claim that may be made by its manufacturer, is not guaranteed or endorsed by the publisher.
