# Supplementary material for: Predicting F v /F m and evaluating cotton drought tolerance using hyperspectral and 1D-CNN
Source: Front Plant Sci. 2022 Oct 18;13:1007150. doi: 10.3389/fpls.2022.1007150 (PMC9623111; doi:10.3389/fpls.2022.1007150)
Supplement: Supplementary file 1 [file DataSheet_1.docx]

***Supplementary Material***

**Supplementary Figures**

**SUPPLEMENTARY FIGURE 1** The experimental layout. CK, normal conditions (blue); DS, drought stress (yellow); 1, Jifeng 554; 2, Jifeng 103; 3, Jifeng 522; 4, Jifeng 908; 5, Jifeng 914; 6, Jifeng 1982; 7, Jifeng 4; 8, 7886; 9, Cangmian 268; 10, Jimian 315; 11, Han 218; 12, Hannong 12; 13, Han 8266; 14, Han 258; 15, Han 686; 16, YM111; 17, Nongda KZ05; 18, Nongdamian 10; 19, Nongdamian 12; 20, Lumianyan 28; 21, Xuzhou 1818; 22, Zhongmiansuo 41; 23, Shandongxiamian11-42; 24, Zhongmiansuo 12; 25, Yumian 19; 26, Ejing 1; 27, Zhongmiansuo 35; 28, Zhongmiansuo 60 ; 29, Xinshi 71143; 30, Xinza 15; 31, Xinshi 17; 32, GK39; 33, 0 shi; 34, Zhongmiansuo 94A915; 35, Lumianyan 36; 36, DP33B; 37, Guoxinmian01; 38, Guoxinmian02; 39, Guoxinmian03; 40, Guoxinmian05; 41, Hanwu 216; 42, Zhongmian 100; 43, Zhongmiansuo 79; 44, Cangmian 666; 45, Han 6203; 46, Shikang 126; 47, Cang 198; 48, Ji 228; 49, Guoxinmian 9; 50, K836; 51, Lumian 522; 52, Lumian 5172; 53, K638; 54, Guoxin 4; 55, Jifeng1187; 56, Jifeng 1458; 57, Jifeng 103; 58, Jifeng 914; 59, Jifeng 965; 60, MH335223; 61, Guoxinmian 11; 62, Zhongmiansuo 17; 63, Chunbeibao; 64, Zhongmiansuo 60;65, CG3020-3; 66, Jimian 2016; 67, Ji 1518; 68, Jihang 8; 69, Jimian 262; 70, Ji 178; 71, Ji 172; 72, Yuzaomian 9110; 73, Dexiamian 1; 74, Jicai 6913; 75, Zhongmiansuo 23; 76, Zhongmiansuo 50; 77, Ji668; 78, Zhibao 86-1; 79, Jimian 958; 80, Jifeng 1271.


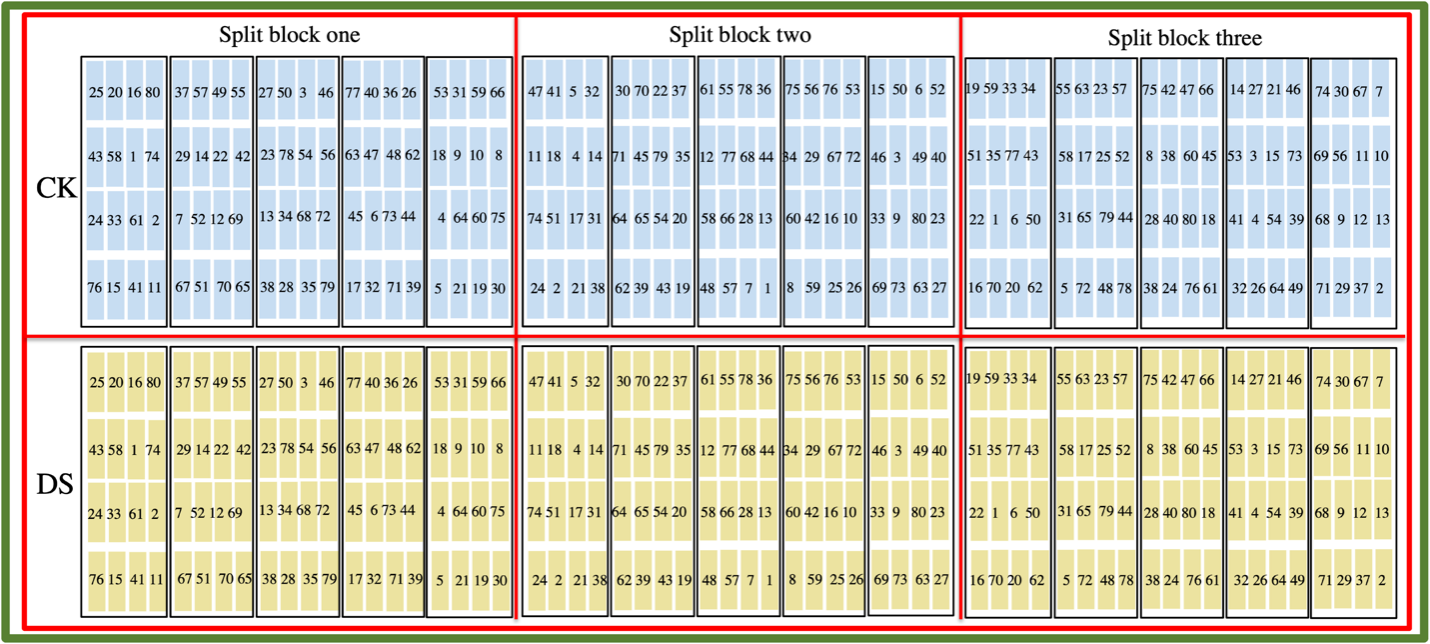


**SUPPLEMENTARY FIGURE 2** Hyperspectral data acquisition using the detailed determination method (A) and leaf selection (B). CK, normal conditions; DS, drought stress.

**
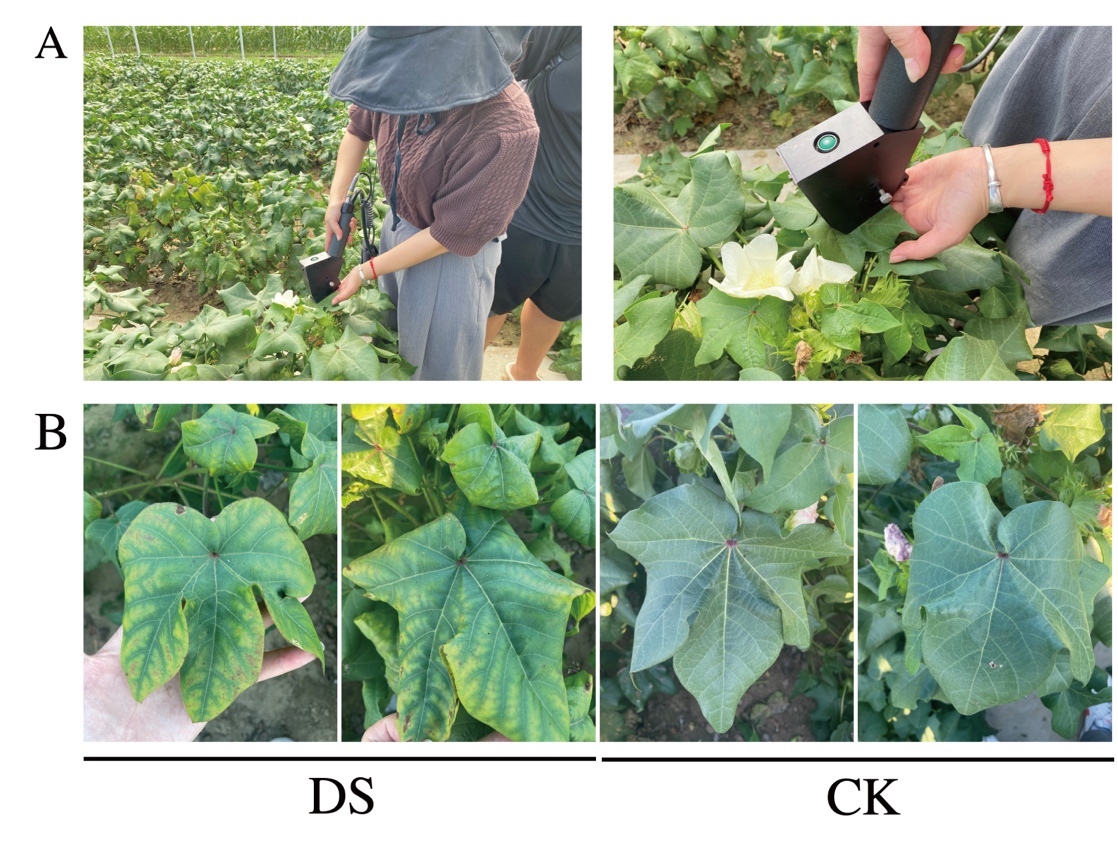
**

**Supplementary Tables**

**SUPPLEMENTARY** **TABLE 1** The different cotton genotypes from the Yellow and Yangtze River basins.

| **No.** | **Cultivar** | **Authorized number** | **No.** | **Cultivar** | **Authorized number** |
| --- | --- | --- | --- | --- | --- |
| 1 | Jifeng 554 | Jishenmian 2009003 | 41 | Hanwu 216 | Jishenmian 2014010 |
| 2 | Jifeng 103 | Jishenmian 20190008 | 42 | Zhongmian 100 | Guoshenmian 2016003 |
| 3 | Jifeng 522 | Jishenmian 20050552 | 43 | Zhongmiansuo 79 | Yushenmian 2010006 |
| 4 | Jifeng 908 |  | 44 | Cangmian 666 | Lushenmian 20160030 |
| 5 | Jifeng 914 | Guoshenmian 2015003 | 45 | Han 6203 | Guoshenmian 2015002 |
| 6 | Jifeng 1982 | Jishenmian 2014001 | 46 | Shikang 126 | Guoshenmian 2008002 |
| 7 | Jifeng 4 | Guoshenmian 20210020 | 47 | Cang 198 | Jishenmian 2007006 |
| 8 | 7886 | GSM08003 | 48 | Ji 228 | Guoshenmian 2008003 |
| 9 | Cangmian 268 | Lushenmian 20160030 | 49 | Guoxinmian 9 | Guoshenmian 2009004 |
| 10 | Jimian 315 | Jishenmian 20190010 | 50 | K836 | Lushenmian 2012018 |
| 11 | Han 218 | Jishenmian 2015003 | 51 | Lumian 522 | Lushenmian 20170041 |
| 12 | Hannong 12 |  | 52 | Lumian 5172 |  |
| 13 | Han 8266 | Guoshenmian 2014001 | 53 | K638 | Lushenmian 2010010 |
| 14 | Han 258 | Jishenmian 2015009 | 54 | Guoxin 4 | Jishenmian 2006008 |
| 15 | Han 686 | Yushenmian 2011010 | 55 | Jifeng1187 | Jishenmian 20200005 |
| 16 | YM111 | Guoshenmian 20170002 | 56 | Jifeng 1458 | Jishenmian 20200004 |
| 17 | Nongda KZ05 | Jishenmian 2013006 | 57 | Jifeng 103 | Guoshenmian 20190014 |
| 18 | Nongdamian 10 | Jishenmian 2015007 | 58 | Jifeng 914 | Guoshenmian 2015003 |
| 19 | Nongdamian 12 | Jishenmian 2014011 | 59 | Jifeng 965 |  |
| 20 | Lumianyan 28 | Guoshenmian 2006012 | 60 | MH335223 | Jishenmian 20190018 |
| 21 | Xuzhou 1818 | I3A01080 | 61 | Guoxinmian 11 | Guoshenmian 2009001 |
| 22 | Zhongmiansuo 41 | Guoshenmian 2002001 | 62 | Zhongmiansuo 17 | xinshemmian19980071991 |
| 23 | Shandongxiamian11-42 |  | 63 | Chunbeibao |  |
| 24 | Zhongmiansuo 12 | Lushenmian 0064 | 64 | Zhongmiansuo 60 | Zheshenmian 2007002 |
| 25 | Yumian 19 | Guoshenmian 2001002 | 65 | CG3020-3 |  |
| 26 | Ejing 1 | GS08002-1991 | 66 | Jimian 2016 | Jishenmian 20199002 |
| 27 | Zhongmiansuo 35 | Guoshenmian 990005 | 67 | Ji 1518 | Jishenmian 2014005 |
| 28 | Zhongmiansuo 60 | Shanshenmian 2013001 | 68 | Jihang 8 | Jishenmian 20190009 |
| 29 | Xinshi 71143 | Guoshenmian 2014004 | 69 | Jimian 262 | Jishenmian 20200002 |
| 30 | Xinza 15 | Jishenmian 2014007 | 70 | Ji 178 | Jishenmian 2015010 |
| 31 | Xinshi 17 | Jishenmian 2015004 | 71 | Ji 172 | Jishenmian 20210003 |
| 32 | GK39 | Guoshenmian 2015008 | 72 | Yuzaomian 9110 | Yushenmian 2012006 |
| 33 | 0 shi |  | 73 | Dexiamian 1 | Lushenmian 0220 |
| 34 | Zhongmiansuo 94A915 | Jinshenmian 2016002 | 74 | Jicai 6913 |  |
| 35 | Lumianyan 36 | Lushenmian 2009022 | 75 | Zhongmiansuo 23 | Guoshenmian 980007 |
| 36 | DP33B |  | 76 | Zhongmiansuo 50 | Guoshenmian 2007013 |
| 37 | Guoxinmian01 | Yushenmian 2009004 | 77 | Ji668 | Guoshenmian 2001001 |
| 38 | Guoxinmian02 |  | 78 | Zhibao 86-1 | GS08009-1984 |
| 39 | Guoxinmian03 | Guoshenmian 2006003 | 79 | Jimian 958 | Guoshenmian 2006005 |
| 40 | Guoxinmian05 | Guoshenmian 2006003 | 80 | Jifeng 1271 | Jishenmian 2012002 |

**SUPPLEMENTARY TABLE 2** Soil properties of the experimental field.

| **Soil properties** | **Content** |
| --- | --- |
| Organic matter | 13.83 g·kg^-1^ |
| Total nitrogen | 0.93 g·kg^-1^ |
| Alkali hydrolyzed nitrogen | 69.45 mg·kg^-1^ |
| Available phosphorus | 17.40 mg·kg^-1^ |
| Available potassium | 121.36 mg·kg^-1^ |

**SUPPLEMENTARY TABLE 3** The characteristic wavelengths of each treatment screened using the continuous projection algorithm (SPA).

| **Period** | **Conditions** | **Characteristics of the wavelength** |
| --- | --- | --- |
| Flowering stage | CK | 1247.9 nm, 1172.5 nm |
|  | DS | 366 nm |
| Boll setting stage | CK | 342.5 nm |
|  | DS | 0 |
| Boll opening stage | CK | 470.6 nm, 708.8 nm |
|  | DS | 1663.6 nm, 341 nm, 1341.4 nm, 993.3 nm, 339.5 nm, 501.5 nm, 1104.2 nm, 695.4 nm |

CK, normal conditions; DS, drought stress.
